# The role of BAFF and APRIL in IgA nephropathy: pathogenic mechanisms and targeted therapies

**DOI:** 10.3389/fneph.2023.1346769

**Published:** 2024-02-01

**Authors:** Chee Kay Cheung, Jonathan Barratt, Adrian Liew, Hong Zhang, Vladimir Tesar, Richard Lafayette

**Affiliations:** ^1^ Division of Cardiovascular Sciences, University of Leicester, Leicester, United Kingdom; ^2^ John Walls Renal Unit, University Hospitals of Leicester National Health Service (NHS) Trust, Leicester, United Kingdom; ^3^ The Kidney & Transplant Practice, Mount Elizabeth Novena Hospital, Singapore; ^4^ Renal Division in the Department of Medicine, Peking University First Hospital, Beijing, China; ^5^ Department of Nephrology, First School of Medicine and General University Hospital, Charles University, Prague, Czechia; ^6^ Department of Medicine, Stanford University, Stanford, CA, United States

**Keywords:** B-cell activating factor BAFF, a proliferation-inducing ligand APRIL, IgA nephropathy, dual inhibition, atacicept

## Abstract

Immunoglobulin A nephropathy (IgAN), characterized by mesangial deposition of galactose-deficient-IgA1 (Gd-IgA1), is the most common biopsy-proven primary glomerulonephritis worldwide. Recently, an improved understanding of its underlying pathogenesis and the substantial risk of progression to kidney failure has emerged. The “four-hit hypothesis” of IgAN pathogenesis outlines a process that begins with elevated circulating levels of Gd-IgA1 that trigger autoantibody production. This results in the formation and deposition of immune complexes in the mesangium, leading to inflammation and kidney injury. Key mediators of the production of Gd-IgA1 and its corresponding autoantibodies are B-cell activating factor (BAFF), and A proliferation-inducing ligand (APRIL), each playing essential roles in the survival and maintenance of B cells and humoral immunity. Elevated serum levels of both BAFF and APRIL are observed in patients with IgAN and correlate with disease severity. This review explores the complex pathogenesis of IgAN, highlighting the pivotal roles of BAFF and APRIL in the interplay between mucosal hyper-responsiveness, B-cell activation, and the consequent overproduction of Gd-IgA1 and its autoantibodies that are key features in this disease. Finally, the potential therapeutic benefits of inhibiting BAFF and APRIL in IgAN, and a summary of recent clinical trial data, will be discussed.

## Introduction

Immunoglobulin A nephropathy (IgAN), characterized by mesangial deposition of immune complexes containing galactose-deficient-IgA1 (Gd-IgA1) and associated autoantibodies, is the most common primary glomerulonephritis worldwide (1–4). However, its true prevalence is uncertain, and estimates are confounded by varying access to healthcare, quality of data capture, and heterogeneity in screening practices and in thresholds to perform a kidney biopsy between centers worldwide (5, 6). Genetic factors are likely to have an important contribution to the variable prevalence observed geographically and between ethnicities (6–8). In North America, IgAN represents approximately 10–20% of biopsy-proven primary glomerulonephritis cases. Prevalence rises to 20–30% in Europe and 40–50% in East Asia (3, 4). Studies spanning multiple countries report an overall incidence of at least 2.5 cases per 100,000/year in adults (9, 10).

IgAN often leads to a progressive reduction in kidney function over several years and is associated with significant morbidity and mortality (11, 12). In approximately 25–50% of patients, IgAN will lead to kidney failure within 10–20 years after diagnosis (11, 13–15). As IgAN is often diagnosed in young adults, many will reach kidney failure in their lifetime, even with current optimal supportive care and available therapy (11, 12, 15). The manifestation of IgAN in the younger population also results in losses to the labor force and a significant social burden (4, 16). In older patients with IgAN, comorbidities such as hypertension, diabetes mellitus, or cardiovascular disease are more likely to exist, and can exacerbate the kidney damage caused by IgAN and complicate its management (17). Late diagnosis in the presence of these comorbidities may further worsen renal prognosis and increase the risk of cardiovascular events (17). Life expectancy in IgAN is reduced by an average of 6 years compared to standardized mortality rates, mainly due to complications of kidney failure (18).

IgAN presents in a diverse array of clinical manifestations, spanning from microscopic to macroscopic hematuria, variable degrees of proteinuria with progressive kidney function decline, to the rare extreme of rapidly progressive glomerulonephritis (RPGN) (14). A kidney biopsy is required for the diagnosis of IgAN, which demonstrates dominant or co-dominant mesangial IgA deposition, and is often accompanied by immunoglobulin G (IgG) and/or immunoglobulin M (IgM) (19). Additionally, there is colocalization of complement 3 (C3) with IgA in >90% of patients, indicative of complement activation. This is thought to occur predominantly via the alternative and/or lectin pathways and is a key mediator of glomerular inflammation and damage (19, 20). The Oxford classification identified several histological features, each independently associated with the risk of kidney disease progression, specifically mesangial hypercellularity, endocapillary hypercellularity, segmental glomerulosclerosis, tubular atrophy/interstitial fibrosis, and the presence of crescents (21). Understanding the interplay between the deposition of Gd-IgA1, complement activation and subsequent glomerular injury may provide further insights into the pathogenesis of IgAN and targets for therapeutic intervention. Proteinuria is the strongest modifiable risk factor for kidney function decline in IgAN (22). Higher levels of circulating Gd-IgA1 and anti-Gd-IgA1 antibodies are also associated with reduced kidney function and, ultimately, kidney failure (23–25).

## Current state of clinical management

Ongoing research into the pathogenesis of IgAN, including genome-wide association studies (GWASs) (6, 7, 26, 27), has revealed insights into its underlying pathogenesis and potential therapeutic targets. It has become clear that personalized treatment is necessary for the optimal management of IgAN given its complexity and variable prognosis. Early assessment is of great importance, as delayed identification may impact prognosis and responsiveness to treatment (28).

Although much progress has been made in understanding the fundamental mechanisms of the pathogenesis of IgAN, there is an unmet need for disease-modifying treatments capable of selectively influencing the synthesis of Gd-IgA1 and/or its associated autoantibodies, both of which play a key role in disease progression (29, 30). The current standard of care for patients with IgAN with proteinuria above 0.5 g/day is supportive treatment with blockade of the renin-angiotensin system (RAS) with either angiotensin-converting enzyme (ACE) inhibitors or angiotensin receptor blockers (ARB), regardless of whether the patient exhibits clinical hypertension (31). Overall cardiovascular risk should also be addressed which includes strict control of blood pressure, dietary sodium reduction, smoking cessation, treatment of hyperlipidemia, weight management, and regular exercise (29, 30).

The use of corticosteroids in the treatment of IgAN is controversial, and there are many uncertainties as to the optimal dosage, duration, and patient selection (32). The Therapeutic Effects of Steroids in IgA Nephropathy (TESTING) study demonstrated that treatment with steroids (6 to 9 months) as compared to placebo reduced kidney function decline, kidney failure, or death due to kidney disease. However, significant adverse events were noted in the TESTING study, as well as several other studies evaluating corticosteroids in IgAN, making long-term use problematic (29, 30, 33–35). Conversely, the STOP-IgAN study provided evidence that affirms the use of supportive care in the treatment of IgAN as opposed to immunosuppression (36). Participants received a comprehensive program of supportive care including dietary sodium reduction, smoking cessation, weight management, and regular exercise. Approximately one-third of the participants, who were initially thought to be candidates for immunosuppression, were discontinued from the study as their levels of proteinuria responded to these measures alone. In addition, in those who were subsequently randomized in the second part of the study, immunosuppression did not significantly affect rates of kidney function decline compared to supportive care (36).

Even with optimized supportive care, a significant proportion of patients will experience ongoing proteinuria and progressive decline of kidney function (29, 30, 35). Long-term results from the STOP-IgAN trial demonstrated that patients with IgAN with persistent proteinuria >0.75 g/day continued to have an unfavorable prognosis (37), with approximately 50% of the participants experiencing death, kidney failure, or greater than 40% decline in estimated glomerular filtration rate (eGFR) over a median duration of 7.4 years (37). Recent large scale epidemiologic data, including a UK renal registry study of 2499 adults with IgAN, demonstrated that the majority of patients are at risk for the development of kidney failure during their lifetime (15).

The current Kidney Disease Improving Global Outcomes (KDIGO) guideline (31) suggests that patients with IgAN who exhibit proteinuria above 1 g/day after receiving 90 days of supportive care could cautiously be considered for corticosteroid therapy, but only after full consideration of the risks weighed against potential benefits. The guideline emphasizes that the clinical benefit of steroid therapy has not been fully established, and patients should first be given an opportunity to participate in a therapeutic clinical trial.

Sodium-glucose cotransporter-2 (SGLT2) inhibitors have emerged as a potential therapeutic option in the management of IgAN (38). The Dapagliflozin and Prevention of Adverse Outcomes in Chronic Kidney Disease (DAPA-CKD) trial provided evidence supporting the use of dapagliflozin in IgAN (39). This trial, which was terminated early due to efficacy, included 270 patients diagnosed with IgAN and demonstrated that the dapagliflozin group experienced fewer primary outcome events, which was a composite of >50% decline in eGFR, end-stage kidney disease, or death from renal or cardiovascular causes over a median follow-up period of 2.1 years. The EMPA-KIDNEY trial confirmed a beneficial effect of the SGLT2 inhibitor empagliflozin in lowering risk of progression of kidney disease or death due to cardiovascular causes among a wide range of patients with CKD (40). These findings were also observed in the 817 patients with IgAN studied as part of EMPA-KIDNEY. Further description of the IgAN cohort is forthcoming (41).

The mechanism of action of SGLT2 inhibitors in IgAN is postulated to be mediated through the reduction of intraglomerular pressure by tubulo-glomerular feedback, as well as other less well-established effects which, in turn, may lead to a reduction in proteinuria and renoprotection (39). The DAPA-CKD trial highlighted a reduction in blood pressure in the dapagliflozin-treated group, and it remains uncertain how much of this blood pressure reduction contributed to the favorable outcomes observed (38). In addition, there was no run-in period where supportive measures were optimized. Therefore, it is unclear how many patients could have responded to these measures alone. The event rate in the placebo-treated group in DAPA-CKD was unusually high. Nevertheless, the use of SGLT2 inhibitors in IgAN as part of supportive care has been adopted widely (38).

Two other therapies have received accelerated approvals by the US Food and Drug administration (FDA) for the treatment of IgAN: targeted release formulation (TRF)-budesonide (Nefecon), which targets the gut mucosal immune system, and sparsentan, a dual endothelin and angiotensin receptor antagonist. Over 20 other agents are currently in clinical development for the treatment of IgAN that target B-cell production of Gd-IgA1, complement activation, and other downstream pathways that are activated following IgA deposition (29).

## Pathogenesis and the four-hit hypothesis

A central feature of IgAN reported in multiple cohorts globally is an increase in circulating levels of Gd-IgA1 (42, 43). The extended hinge region of IgA1 is sequentially glycosylated by the addition of *O*-glycans containing N-acetylgalactosamine (GalNAc) and terminal galactose residues under the influence of glycosyltransferases, as a post-translational modification process. In patients with IgAN, there is an increase in IgA1 that lacks galactose residues from its hinge region (termed galactose-deficient IgA1 or Gd-IgA1), leading to exposure of GalNAc residues. These GalNAc residues may be recognized by anti-glycan autoantibodies in susceptible individuals, resulting in the formation of circulating immune complexes (44). These immune complexes have a high affinity for mesangial cells and, once deposited in the mesangium, can trigger a series of inflammatory responses.

**Figure 1:**
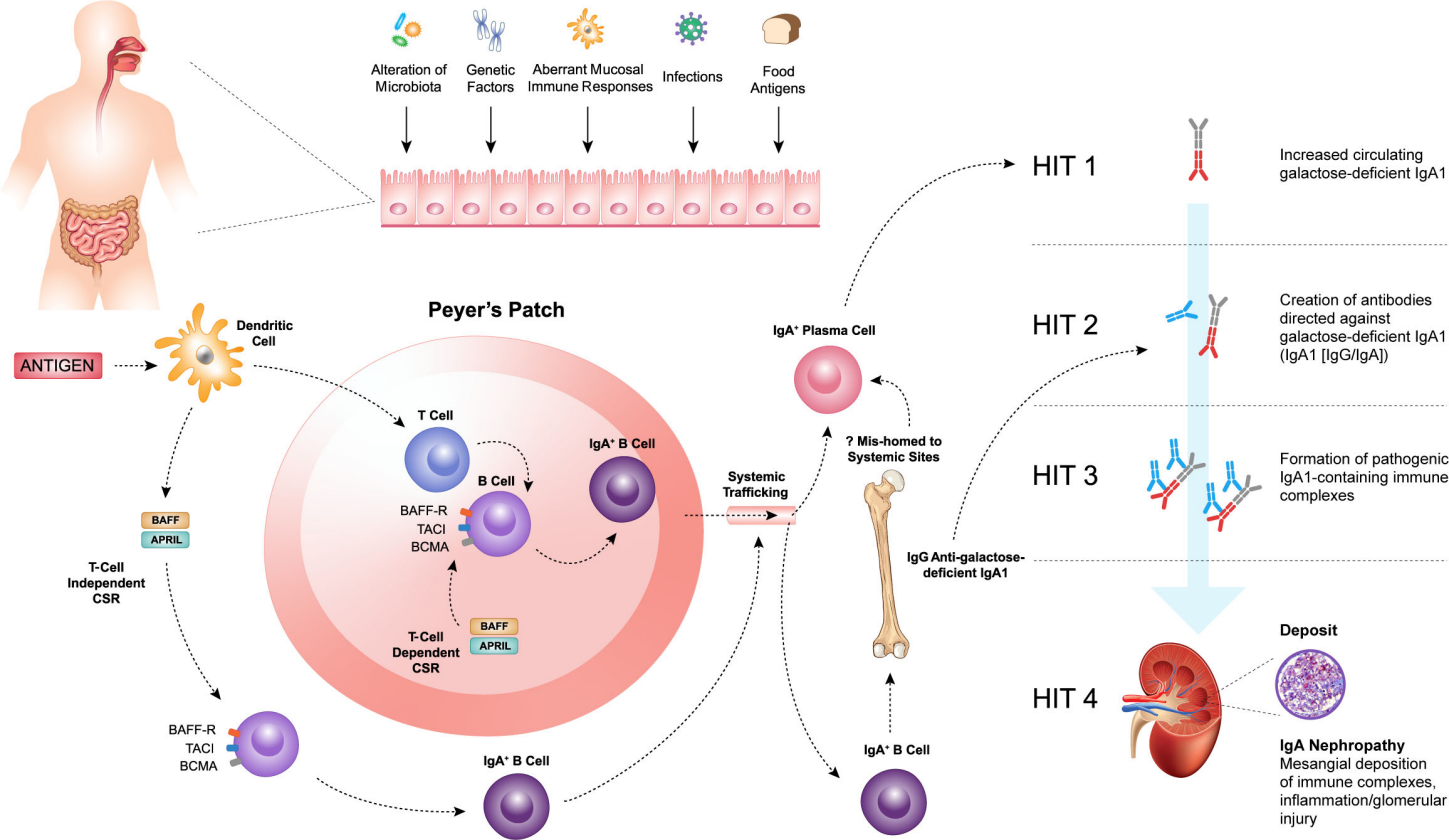
Within the mucosal-associated lymphoid tissue, antigens are taken up by antigen-presenting cells, such as dendritic cells, resulting in naïve B-cell activation and class switch recombination (CSR) to form committed IgA^+^ B cells, which require a T-cell dependent (TD) or T-cell independent (TID) co-stimulatory signal. B-cell activating factor (BAFF) and a proliferation-inducing ligand (APRIL), members of the tumor necrosis factor superfamily, play a key role in driving both TD and TID CSR and, thus, production of IgA^+^ B cells. The pathogenesis of IgAN is tied to aberrations in B-cell activation and CSR that lead to the increased production of galactose-deficient IgA1 (Gd-IgA1) (Hit 1) and the subsequent synthesis of autoantibodies directed against Gd-IgA1 (Hit 2). The formation of pathogenic IgA1-containing immune complexes (Hit 3) provokes inflammation leading to glomerular injury (Hit 4).

Several lines of evidence support a mucosal source for the increased circulating Gd-IgA1 in IgAN (2, 45–47; **Figure 1**). The majority of human IgA is produced by plasma cells residing in mucosal-associated lymphoid tissue (MALT), and two major regions are implicated in IgAN: the gut-associated lymphoid tissue (GALT) and nasopharynx-associated lymphoid tissue (NALT) (46, 48). The mucosal-kidney connection is evident in patients with IgAN where hematuria may occur in close temporal association with mucosal infections, such as tonsillitis or other upper respiratory tract infections and gastrointestinal infections (45, 48).

Within the MALT, antigens are taken up by antigen-presenting cells, such as dendritic cells (DCs) resulting in naïve B-cell activation and class switch recombination (CSR) to form committed IgA^+^ B cells, which require a T-cell dependent (TD) or T-cell independent (TID) co-stimulatory signal (**Figure 1**) (49, 50). In the context of normal mucosal immunity, the switch to IgA production is of paramount importance. IgA serves as the first line of defense at mucosal surfaces, neutralizing pathogens while maintaining homeostasis with host commensal organisms. However, the pathogenesis of IgAN is intricately tied to aberrations in B-cell activation and CSR that lead to the increased production of Gd-IgA1.

B-cell activating factor (BAFF) and a proliferation-inducing ligand (APRIL), members of the tumor necrosis factor (TNF) superfamily, play a key role in driving both TD and TID CSR and, thus, production of IgA^+^ B cells (49–53). The IgA^+^ B cells then leave the MALT and travel to effector sites via the lymphatic system and circulation. It has been hypothesized that in patients with IgAN, altered homing leads to mucosally-derived IgA^+^ plasma cells being mis-trafficked to systemic sites, such as the bone marrow, resulting in the production of “mucosal-type” Gd-IgA1 in the circulation (54–56). Other recent work has highlighted possible roles for reverse transcytosis of Gd-IgA1 from the gut lumen, or trafficking of IgA^+^ B cells to the kidneys in the pathogenesis of IgAN (57, 58).

Irrespective of its exact source, raised Gd-IgA1 levels are not sufficient to induce IgAN, as first-degree relatives of patients with IgAN may also have similarly elevated levels of Gd-IgA1 and not develop kidney disease (59, 60). Initiation of kidney injury associated with IgAN requires the formation of immune complexes including Gd-IgA1 (61). These and other observations provide the conceptual framework for the proposed four-hit hypothesis of the pathogenesis of IgAN. This widely accepted hypothesis proposes that increased levels of circulating Gd-IgA1 (Hit 1) induce the production of IgA and IgG autoantibodies (Hit 2), resulting in the formation of immune complexes (Hit 3), that are then deposited in the glomeruli and induce kidney injury and damage (Hit 4) (**Figure 1**) (42). Therefore, IgAN should be considered an autoimmune disease (42, 61, 62). Activation of mesangial cells, production of extracellular matrix, activation of the complement system, and the subsequent release of cytokines and chemokines lead to local inflammation and fibrosis. These, in turn, cause glomerular injury, disruption of the glomerular filtration barrier, and ultimately hematuria, proteinuria, and progressive kidney dysfunction, which are characteristic features of IgAN.

Multiple therapeutics under development target different aspects of IgAN pathobiology from the upstream plasma cells that produce immune complexes to the downstream components of the complement system (63).

## Targeting B cells in the treatment of IgAN

Central to the formation of circulating immune complexes in IgAN are the B cells responsible for producing its components, specifically Gd-IgA1 and IgG/IgA anti-glycan autoantibodies. Critical factors that influence B-cell activity are the B-cell survival mediators, BAFF and APRIL, members of the TNF superfamily that share considerable homology and act via a shared set of B-cell receptors. These cytokines have emerged as important therapeutic targets to potentially impact formation of IgA, Gd-IgA1 and immune complexes, and therefore also alter the progression of IgAN (45, 49).

**Figure 2:**
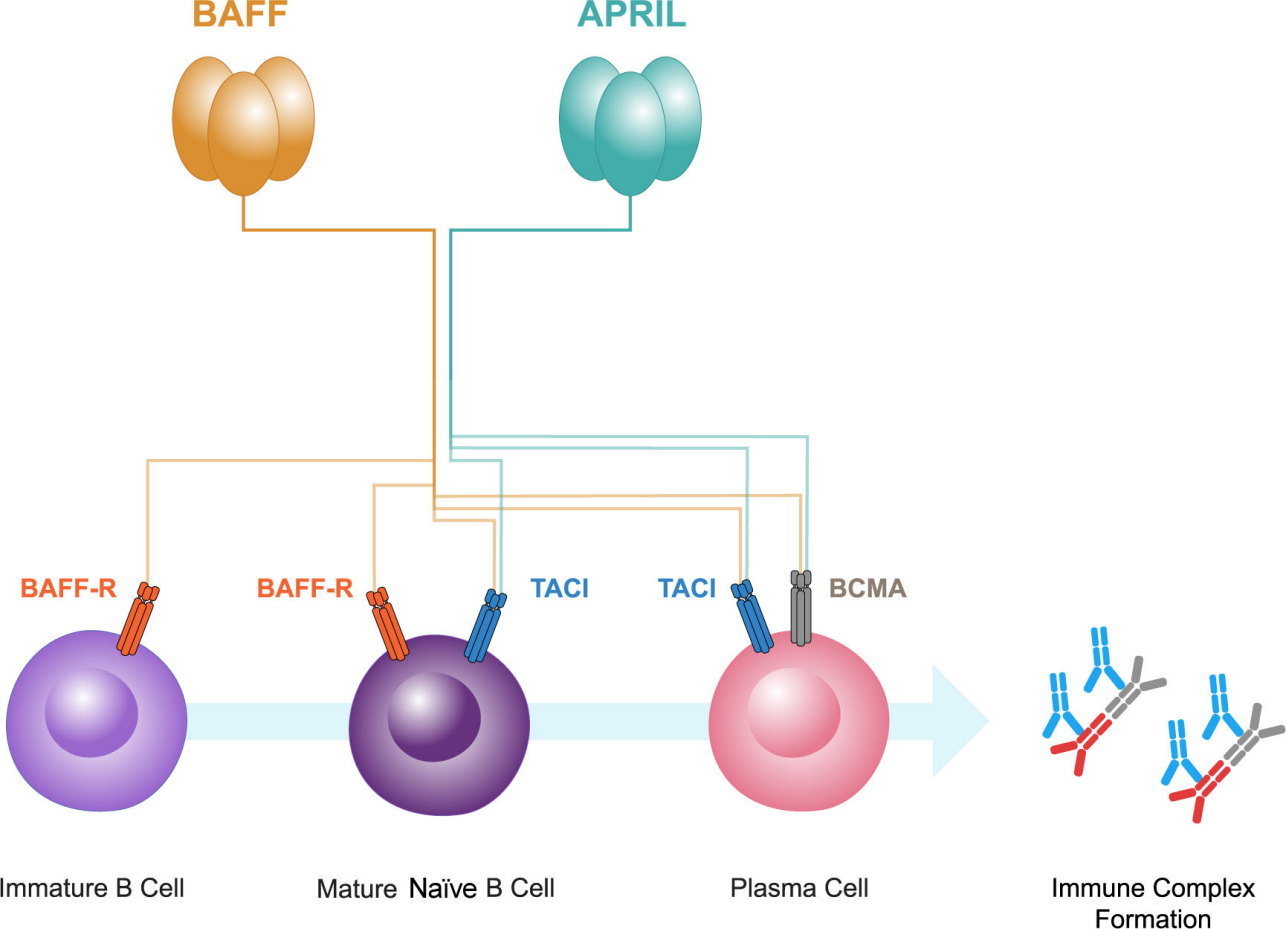
B-cell activating factor (BAFF) and a proliferation-inducing ligand (APRIL), members of the tumor necrosis factor superfamily, both bind to two receptors, B-cell maturation antigen (BCMA) and transmembrane activator and calcium-modulator and cytophilin ligand interactor (TACI). BCMA is primarily expressed on plasma cells, whereas TACI is expressed on mature B cells and plasma cells. A third receptor, BAFF receptor (BAFF-R), that is specific for BAFF, is expressed mainly on immature and mature naive B cells. BAFF and APRIL have pivotal roles in the interplay between gut mucosal hyper-responsiveness, B cell activation, and the consequent overproduction of Gd-IgA1 and its autoantibodies resulting in immune complex formation.

BAFF and APRIL both bind to two receptors, B-cell maturation antigen (BCMA) and transmembrane activator and calcium-modulator and cyclophilin ligand interactor (TACI) (52, 53; **Figure 2**). BCMA is primarily expressed on plasma cells, whereas TACI is expressed on mature B cells and activated plasma cells. A third receptor, BAFF receptor (BAFF-R), that is specific for BAFF, is expressed mainly on B cells (52, 53, 64, 65). BAFF-R is also expressed on human mesangial cells and tubular epithelial cells (TECs) (66–68). BAFF causes increased BAFF-R expression in human TECs, implicating an autocrine loop (67).

**Figure 3:**
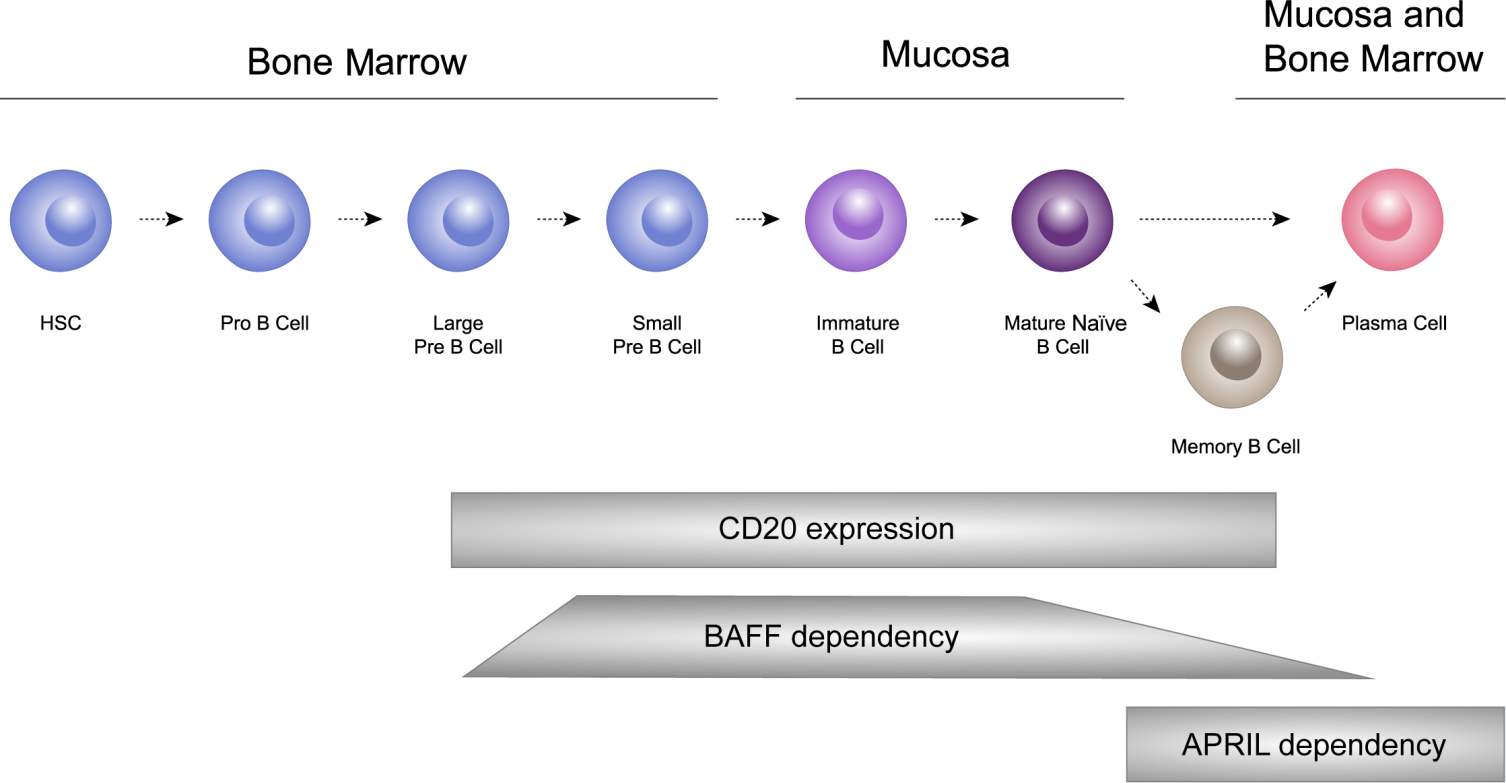
B cells undergo a sequential process of maturation and selection and play a crucial role in the adaptive immune system by generating antibodies as a response to foreign antigens. B cells have the ability to differentiate into short-lived plasma cells/plasmablasts, and long-lived plasma cells, which are responsible for producing immunoglobulins, including IgA. HSC: hematopoietic stem cells.

B cells undergo a sequential process of maturation and selection and play a crucial role in the adaptive immune system by generating antibodies as a response to foreign antigens (69). B cells have the ability to differentiate into short-lived plasma cells/plasmablasts, and long-lived plasma cells, which are responsible for producing immunoglobulins, including IgA (53, 70, 71). In healthy individuals, memory B cells and plasma cells comprise important and independently regulated components of immunologic memory (72; **Figure 3**).

BAFF is instrumental in B-cell maturation. It is vital for the activation, survival and differentiation of peripheral B cells. The interaction of BAFF with its primary receptor, BAFF-R, is crucial for maintaining B-cell homeostasis (73). The activation of B cells requires antigen presentation and a costimulatory signal via TD or TID mechanisms (49, 50, 74). Microbial antigens containing pathogen-associated molecular patterns (PAMPs) may induce the release of BAFF and APRIL from antigen presenting cells such as dendritic cells, by binding to pattern recognition receptors such as Toll-like receptors on their cell surface, therefore linking innate and adaptive immune responses (75). BAFF and APRIL are then able to activate B cells via their receptors BAFF-R, BCMA, and TACI (see **Figure 2**) (52, 53). The activation of these receptors facilitates the process of IgA CSR and B-cell survival (52, 75), promoting the microbial-driven effects on IgA B-cell responses in the mucosa (76).

BAFF supports the survival and differentiation of B cells into long-lived plasma cells that reside within survival niches in the bone marrow, secondary lymphoid organs and mucosal sites (77, 78). These plasma cells can continuously produce antibodies, including Gd-IgA1, without the need for further stimulation. By supporting TD and TID CSR to IgA and IgG and the differentiation and survival of these B cells, BAFF may contribute to the sustained production of Gd-IgA1 and anti-Gd-IgA1 autoantibodies in IgAN.

APRIL is also crucial for B-cell survival and differentiation (53, 70). Through its receptors, APRIL can influence the differentiation of B cells into plasma cells (53, 70, 71). Both Gd-IgA1 and anti-Gd-IgA1 autoantibody production are believed to be driven primarily by mucosal plasma cells that contribute to autoimmune disorders by producing high quantities of autoantibodies (24, 79, 80). APRIL can stimulate the production of IgA by B cells. In the mucosal environment, where IgA production is predominant out of all immunoglobulin subclasses, APRIL, like BAFF, can induce naive B cells to undergo class switching to IgA-producing B cells through signaling via TACI and/or BCMA (75). *In vitro* studies have demonstrated that when cultured B cells from IgAN patients are stimulated with APRIL, there is an increased production of Gd-IgA1, suggesting a direct role in promoting the aberrant glycosylation of IgA1 (81) (see **Figure 1**).

Given their pivotal roles in the production of Gd-IgA1 and their autoantibodies, targeting BAFF and APRIL offers a logical therapeutic strategy for IgAN. Extensive preclinical and clinical studies have been conducted as to the potential efficacy of inhibition of BAFF and/or APRIL.

## Preclinical and clinical evidence for the role of BAFF in IgAN

### Preclinical evaluation of the role of BAFF

Transgenic mice overexpressing BAFF (BAFF-Tg) exhibit several notable immunological changes compared to wild-type mice. One of the most prominent observations is elevated serum levels of IgA (71). This increase in IgA is accompanied by an increased number of IgA^+^ plasma cells in the gut lamina propria. Deposition of IgA immune complexes in the glomerular mesangium, in a similar manner to human IgAN, is also observed. These mice demonstrated an expanded B-cell compartment which may contribute to autoimmunity. Additionally, BAFF-Tg mice demonstrate excessive numbers of mature B cells, spontaneous germinal center reactions, secretion of autoantibodies, high plasma cell numbers in secondary lymphoid organs, elevated numbers of effector T cells, and Ig deposition in the kidneys (73, 82). These results support that BAFF alone can directly stimulate B-cell proliferation. The overexpression of BAFF also increased B-cell viability, via BAFF-R.

In further studies of this model (76), BAFF-Tg mice had increased levels of aberrantly glycosylated polymeric IgA, mesangial IgA deposits and expanded mesangial matrix. BAFF-Tg mice also developed IgA-dependent hematuria and proteinuria. Commensal bacteria were required for the development of the IgAN-like phenotype. BAFF-Tg mice raised in germ-free environments that prevented gut colonization by commensal bacteria were protected from the development of elevated serum IgA, renal IgA deposits, IgA^+^ plasma cells in the gut and presence of commensal-bacteria reactive IgA antibodies in the bloodstream, but these occurred when commensal bacteria were re-introduced.

Currie and colleagues (57) characterized the immune responses to mucosal microbiota in BAFF-Tg mice. Mice nasally infected with *Neisseria meningitidis* (Nme) had increased levels of circulating Nme-specific IgA with increases in mesangial expansion and IgA deposition. Interestingly, an increase in anti-Nme IgA^+^ secreting cells was observed in kidney tissue, suggesting that these cells migrated there following priming from a mucosal source, and could contribute locally to IgA production and its glomerular deposition in IgAN. These observations together suggest that perturbations to the mucosal microbiota and increased reactivity of B cells driven by high levels of BAFF could both contribute to the IgAN-like features in this model.

### Clinical evidence of the role of BAFF

The central role of BAFF in B-cell proliferation, differentiation, and production of IgA1 and associated autoantibodies suggests that it has an important clinical role in IgAN (71, 83). Multiple studies have demonstrated that serum levels of BAFF are elevated in IgAN patients compared to healthy controls (49, 76, 84, 85). A positive correlation has been observed between serum levels of BAFF and IgA1 levels in IgAN patients (84). In addition, BAFF appears to be associated with the severity of IgAN, both histologically and clinically (84). Serum levels of BAFF have been positively correlated with mesangial IgA deposition density (85), and presence of mesangial hypercellularity, segmental glomerulosclerosis and interstitial fibrosis/tubular atrophy (84). Circulating BAFF levels have been associated with reduced kidney function in patients with IgAN, in terms of reduced eGFR and increased serum creatinine (84). Additional studies have demonstrated that tonsillar mononuclear cells from patients with IgAN can be induced to express increased levels of BAFF and IgA as compared to cells from non-IgAN controls (86, 87).

The efficacy and safety of blisibimod, a selective inhibitor of BAFF, was evaluated in patients with IgAN in a Phase 1/2 study known as BRIGHT-SC (NCT02062684). Preliminary findings were presented in 2016 that indicated a significant reduction in B-cell subsets and Ig levels within the blisibimod group, providing evidence of the pharmacological suppression of BAFF (88). Proteinuria levels remained stable with blisibimod, while a marked worsening was observed in those treated with placebo, although full results from this study have not been published to date. The long-term effectiveness of targeting BAFF alone in IgAN are unknown.

## Preclinical and clinical evidence for the role of APRIL in IgAN

### Preclinical evidence of the role of APRIL

Mouse models have also provided insights into the potential role of APRIL in IgAN. APRIL-deficient mice display impaired IgA class switching, defective IgA antibody responses in response to mucosal immunization and markedly decreased numbers of IgA^+^ plasma cells in the lamina propria of the small intestine (89).

The role of APRIL inhibition has been studied in the grouped ddY mouse model of IgAN (90). These studies demonstrated that anti-APRIL treatment significantly decreased albuminuria, serum IgA levels, and glomerular IgA deposition. These results suggest that the APRIL signaling pathway is also implicated in the production of pathogenic IgA in this model (34).

Myette et al. (91) evaluated the effectiveness of APRIL inhibition in the grouped ddY (gddY) mouse model of IgAN. Treatment of gddY mice with a mouse anti-APRIL monoclonal antibody led to lower serum IgA levels, reduced circulating immune complexes, reduced IgA, IgG, and C3 glomerular deposits, and a reduction in proteinuria.

### Clinical evidence of the role of APRIL

Multiple observations support the role of APRIL in the pathogenesis of IgAN. A polymorphism in the gene encoding APRIL (TNFSF13) and the TACI receptor (TNFRSF13B) confer susceptibility to IgAN (8, 49, 92). Increased APRIL levels have been demonstrated in patients with IgAN and correlate with disease severity (proteinuria and eGFR) and increased expression of Gd-IgA1 (81). Additionally, higher levels of APRIL are associated with a worse prognosis in IgAN (93). Therapeutic strategies inhibiting APRIL have the potential to limit IgA and, therefore, Gd-IgA1 production by autoimmune-associated plasma cells (63).

The anti-APRIL antibody, VIS649 (sibeprenlimab), has been studied in healthy volunteers and patients with IgAN. In healthy volunteers, treatment with VIS649 led to suppression of serum free APRIL levels, and reductions in serum Gd-IgA1, IgA, IgM, and to a lesser extent IgG. The authors note that median changes of BAFF levels from baseline were less than 35% (94). A Phase 2 study of sibeprenlimab has been completed which randomized 155 patients with IgAN to receive monthly intravenous (IV) sibeprenlimab doses at 2, 4, or 8 mg/kg or placebo for 12 months (95). This study demonstrated that treatment with sibeprenlimab reduced Gd-IgA1 and IgA levels with associated significant reductions in proteinuria compared to placebo, with reductions in 24-hour urinary protein to creatinine ratio (UPCR) at 12 months of 47.2%, 58.8%, and 62.0% in the 2, 4, or 8 mg/kg IV doses respectively, compared to a 20.0% reduction in the placebo group. The change in eGFR at 12 months was -2.7, +0.2, and -1.5 mL/min/1.73m^2^, in the sibeprenlimab 2, 4, and 8 mg/kg groups respectively, compared to a -7.4 mL/min/1.73m^2^ decrease in the placebo group, suggesting that treatment with sibeprenlimab slowed the decline of kidney function. No safety concerns were noted with sibeprenlimab treatment. A Phase 3 trial of sibeprenlimab in IgAN is in progress (VISIONARY; https://clinicaltrials.gov/study/NCT05852938).

Interim results from an open-label single arm Phase 1/2 study evaluating another anti-APRIL monoclonal antibody, BION-1301 (zigakibart), in patients with IgAN also demonstrated sustained reductions in levels of Gd-IgA1, IgA and to a lesser extent IgG, which was accompanied by a reduction in proteinuria maintained throughout the study (96). Zigakibart is also now being studied in a global Phase 3 clinical trial (BEYOND; https://clinicaltrials.gov/study/NCT05852938).

## The role of BAFF and APRIL in IgAN and the rationale for dual inhibition

Current preclinical and clinical evidence support a potential role for both BAFF and APRIL on the generation and survival of plasma cells producing both Gd-IgA1 and anti-Gd-IgA1 autoantibodies (53, 54, 76–78, 97, 98). BAFF and APRIL each mediate signals to their cognate receptors that result in the activation, differentiation and survival of pathogenic plasma cells in IgAN patients. This suggests that dual inhibition may be beneficial (99, 100). Whether long-term sole inhibition of APRIL or BAFF alone could result in unintended compensatory upregulation of the other pathway in patients with IgAN is unknown. A report of a rare case of common variable immunodeficiency and complete deficiency in APRIL demonstrated elevated levels of BAFF in the circulation (101). Similarly, a human monocyte cell line lacking BAFF displayed increased levels of APRIL (102).

Both BAFF and APRIL play crucial roles in B-cell homeostasis and function (52, 78, 97). In the context of IgAN, the dysregulation of BAFF and APRIL, and their signaling pathways, may contribute to the pathogenesis of the disease by affecting B cell and plasma cell function, thereby influencing IgA and anti-glycan autoantibody production, immune complex formation and their subsequent deposition in the kidneys (76, 103). BAFF is primarily involved in the survival and maturation of B cells (97). It supports the survival of transitional and mature B cells, and is crucial for B-cell maturation into immunoglobulin-producing cells and also has a role in their survival (52, 78, 97). APRIL has a more defined role in the later stages of B-cell differentiation and is also important for the survival of long-lived plasma cells and other B-cell subsets (78). The significance of this process is particularly pronounced within the bone marrow and MALT where enduring plasma cells reside, generating antibodies (see **Figure 3**).

The interaction of APRIL with its receptors TACI, and especially BCMA, facilitates plasma cell survival. Additionally, BAFF has been shown to selectively enhance the survival and effector function of CD38+ plasmablasts generated from activated human memory B cells (104). Plasma cells are characterized by high cell surface expression of CD38 and loss of CD20 (72). This may provide a rationale for the lack of efficacy in IgAN of rituximab that targets CD20+ B cells (30, 63, 72, 105). In a randomized controlled trial, despite adequate depletion of peripheral B cells, treatment of patients with IgAN with rituximab did not reduce serum levels of Gd-IgA1 or anti-Gd-IgA1 autoantibodies. In addition, no clinical benefit in terms of proteinuria or kidney function was observed compared with standard therapy (106).

As noted, current supportive treatments for IgAN such as use of renin-angiotensin inhibitors are aimed at slowing disease progression, and are not specific for IgAN. The dual inhibition of BAFF and APRIL offers a targeted approach that addresses the underlying immunological basis of the disease, potentially providing a disease-modifying therapeutic strategy that could slow or halt IgAN progression. Although long term data regarding the clinical impact of APRIL and/or BAFF inhibition are not yet available, dual inhibition of BAFF and APRIL in a preclinical lupus model appeared to be optimal in comparison to BAFF inhibition alone, with regards to preventing development of proteinuria, reducing plasma cell numbers, and reducing the production of autoantibodies (anti-double stranded deoxyribonucleic acid [DNA] antibodies) (107).

A novel biological agent that targets both BAFF and APRIL, and suppresses B-cell-mediated autoimmune responses, has been approved for use in systemic lupus erythematosus in China (108, 109). Telitacicept contains the Fc portion of IgG1 combined with the extracellular soluble portion of TACI (110). Results from a recent clinical trial in China in IgAN patients indicate that telitacicept reduced proteinuria and was well tolerated (33). Another dual inhibition agent, povetacicept, was shown to inhibit BAFF and APRIL in a mouse lupus nephritis model, with significant reductions in serum IgM, IgA, and IgG levels after a single dose (111). Povetacicept is currently being evaluated in an open-label Phase 2 study in adult patients with IgAN, membranous nephropathy, or lupus nephritis to determine its safety, efficacy, and optimum dose in these autoimmune renal diseases (https://clinicaltrials.gov/study/NCT05732402).

Atacicept is a novel immunomodulatory therapy composed of a fully humanized recombinant fusion protein consisting of the Fc region of human IgG1 and the binding portion of TACI (99, 100, 112). Atacicept acts by binding soluble BAFF and APRIL, and membrane-bound BAFF, interfering with the cellular interactions of these cytokines and their receptors. Atacicept has been studied in over 1500 patients, with no concerning safety signals to date (113).

The JANUS trial was a Phase 2a, multinational, randomized, double-blind, placebo-controlled study to investigate the safety and efficacy of atacicept in patients with IgAN and persistent proteinuria (100). The study enrolled a total of 16 patients, with 5 receiving placebo, 6 receiving atacicept 25 mg, and 5 receiving atacicept 75 mg for up to 72 weeks. Atacicept demonstrated a safety profile that was comparable to placebo, with no new safety signals identified. Importantly, there was no increase in severe TEAEs, including severe hypogammaglobulinemia, with long-term atacicept exposure. Atacicept treatment led to dose-dependent reductions in serum IgA, IgG, and IgM. Moreover, substantial reductions in the levels of Gd-IgA1 were observed, with changes of -25% and -60% at week 24 for atacicept 25 mg and 75 mg, respectively. A clinically meaningful reduction in 24-hour proteinuria was observed with atacicept. While there was a decline in renal function by eGFR in the placebo group, both doses of atacicept resulted in stabilization of eGFR, with improvements observed up to week 72 (100).

Based on these positive findings, the Phase 2b ORIGIN study was initiated (114). ORIGIN completed the global randomized, double-blind, placebo-controlled portion of the Phase 2b trial that enrolled 116 IgAN participants. The primary endpoint was met with a mean UPCR that was reduced from baseline by 31% in the pooled atacicept 150 mg and 75 mg arms compared with a 7% reduction from baseline in the placebo arm (Δ=25%, p=0.037) at 24 weeks. The atacicept 150 mg arm achieved a 33% proteinuria reduction from baseline at Week 24 and was the only individual treatment arm that showed a statistically significantly greater reduction than placebo (Δ=28%, p=0.047). At 36 weeks, there was a decrease from baseline in mean UPCR of 33% in the atacicept 150 mg arm compared to a 3% increase in the placebo arm, resulting in a 35% reduction at week 36 with atacicept 150 mg versus placebo (p=0.012). Results of the intent-to-treat (ITT) analysis are supported by a prespecified per-protocol (PP) analysis (n=26 atacicept 150 mg; n=26 placebo), where the atacicept 150 mg arm showed a 40% reduction from baseline in UPCR at 36 weeks compared with a 5% reduction in the placebo arm (Δ=43%, p=0.003). At week 36, mean eGFR increased from baseline by 1.6% in the atacicept 150 mg arm compared with an 8.5% reduction from baseline in the placebo arm, resulting in an 11% difference (p=0.038). The difference in the adjusted geometric mean change in eGFR at week 36 was 5.8 mL/min/1.73m^2^ in the atacicept 150 mg arm versus placebo. In addition, the atacicept 150 mg arm achieved a 64% reduction from baseline at week 36 in Gd-IgA1 (p<0.0001) (114).

The safety results indicated that atacicept was generally well-tolerated with no increased rate of infections compared to placebo, a low rate (2%) of serious AEs overall with none in the atacicept 150 mg group, and no study drug discontinuation or interruptions due to hypogammaglobulinemia (114).

## Conclusions

The findings from the various preclinical and clinical studies discussed underscore the potential importance of BAFF and APRIL in the modulation of the immune system, and particularly in the immune dysregulation that is observed in IgAN. The early studies supporting the safety and efficacy of inhibiting these cytokines open a promising avenue for the treatment of IgAN, showcasing a dose-dependent reduction in serum IgA levels alongside substantial reductions in the levels of Gd-IgA1. Results from Phase 2 IgAN trials support the potential benefits of dual inhibition of BAFF and APRIL in IgAN, and ongoing Phase 3 clinical studies will shed light on how this approach may offer greater and/or more sustained efficacy in preserving kidney function and preventing kidney failure in patients with IgAN.

## Author contributions

CC: Writing – review & editing. JB: Writing – review & editing. AL: Writing – review & editing. HZ: Writing – review & editing. VT: Writing – review & editing. RL: Writing – review & editing.
